# Detection and quantification of *Flavobacterium psychrophilum* in water and fish tissue samples by quantitative real time PCR

**DOI:** 10.1186/1471-2180-14-105

**Published:** 2014-04-26

**Authors:** Nicole Strepparava, Thomas Wahli, Helmut Segner, Orlando Petrini

**Affiliations:** 1 Laboratory of Applied Microbiology, University of Applied Sciences and Arts of Southern Switzerland, Via Mirasole 22a, 6500 Bellinzona, Switzerland; 2 Centre for Fish and Wildlife Health, University of Bern, Länggassstrasse 122, 3001 Bern, Switzerland; 3 POLE Pharma Consulting, Breganzona, Switzerland

## Abstract

**Background:**

*Flavobacterium psychrophilum* is the agent of Bacterial Cold Water Disease and Rainbow Trout Fry Syndrome, two diseases leading to high mortality. Pathogen detection is mainly carried out using cultures and more rapid and sensitive methods are needed.

**Results:**

We describe a qPCR technique based on the single copy gene β’ DNA-dependent RNA polymerase (rpoC). Its detection limit was 20 gene copies and the quantification limit 10^3^ gene copies per reaction. Tests on spiked spleens with known concentrations of *F. psychrophilum* (10^6^ to 10^1^ cells per reaction) showed no cross-reactions between the spleen tissue and the primers and probe. Screening of water samples and spleens from symptomless and infected fishes indicated that the pathogen was already present before the outbreaks, but *F. psychrophilum* was only quantifiable in spleens from diseased fishes.

**Conclusions:**

This qPCR can be used as a highly sensitive and specific method to detect *F. psychrophilum* in different sample types without the need for culturing. qPCR allows a reliable detection and quantification of *F. psychrophilum* in samples with low pathogen densities. Quantitative data on *F. psychrophilum* abundance could be useful to investigate risk factors linked to infections and also as early warning system prior to potential devastating outbreak.

## Background

Flavobacteria are non-fermentative, catalase and oxidase positive, gram negative, yellow rods frequently isolated from different ecosystems [1-3]. Some species, in particular *Flavobacterium branchiophilum, F. columnare* and *F. psychrophilum* are feared fish pathogens responsible for disease outbreaks in fish farms worldwide [4-9]. *F. psychrophilum* cause either skin, gills and fin lesions as well as systemic disease in internal fish organs, the so called Bacterial Cold Water disease (BCW) and Rainbow Trout Fry Syndrome (RTFS), which can both lead to high mortality in the populations affected [4,10].

Diagnosis of *F. psychrophilum* infections relies mainly on macroscopic symptoms, microscopic examination of fresh samples of fish spleens, and cultures of samples from tissues on non-selective agar medium [11-14]. Due to the often only superficial location of the disease on the fish as well as low densities and slow growth of the pathogen, early stages of infection are easily overlooked. This can lead to false negative results, thus increasing the number of incorrect diagnoses [15].

Fluorescent in situ hybridization (FISH) has recently been described to diagnose *F. psychrophilum* infections in fish: the method is fast, reliable, and allows detection of *F. psychrophilum* concentrations of >10^5^ cells/ml in water and spleen samples [16]. In some cases FISH provide quantitative results [17], but this *F. psychrophilum* specific FISH, allows only a qualitative detection but no quantification of the pathogen [16].

In the past few years, PCR methods have been described to detect and diagnose *F. psychrophilum* infections [18,19]. PCR, as well as nested PCR, are highly sensitive, fast, and could allow simultaneous detection of different pathogens [20,21]. Currently available PCR techniques can be used to detect *F. psychrophilum* in a sample [18,19].

Real time quantitative PCR (qPCR) has been used in several studies to improve sensitivity of methods of detection and quantification of bacteria [22]. Due to its high sensitivity, this technique has widely been used to discover low amounts of pathogen DNA in the environment or in an organism during infection, to monitor its spread as well as to study healthy carriers as pathogen reservoirs [22-24]. Recently two qPCR for *F. psychrophilum* were developed [25,26] both however were tested only on fish tissues and there is still the need for quantitative methods allowing quantification of *F. psychrophilum* in field samples such as water and soil.

The choice of a species-specific marker gene is crucial for a good diagnostic PCR. *rpoC*, a single copy gene present in *Flavobacterium* spp., has been used to assess phylogenetic relationships and mutation rates in different genera and species and has been shown to be more variable at the interspecific level than the 16S rRNA gene [27-29]. Moreover, each bacterial cell may contain a variable number of 16S rRNA genes copies. For instance, *F. psychrophilum* harbors on average 6 16S rRNA genes copies, thus making it difficult to precisely quantify the number of bacteria in a sample [26,30]. Therefore, targeting single copy genes allows a straightforward and more accurate quantification of the pathogen, with one gene copy corresponding to one bacterial cell [31]. In addition, *rpoC* variability could provide specific amplification of the *F. psychrophilum* target sequence, making *rpoC* a good candidate for use in qPCR.

Therefore, the aim of this study was to develop a qPCR using the rpoC gene as a target to rapidly detect and quantify *F. psychrophilum* in the natural environment.

## Results

All *F. psychrophilum* (100 isolates) were correctly detected with the primers used while all other 130 strains were not amplified (Table 1). The specific primers used in this study showed excellent specificity, sensitivity, and positive and negative predicted values (all 100%).

**Table 1:** **Bacteria used to test specificity and sensitivity of *F. psychrophilum *** **specific rpoC primers**

**Taxon**	**No. of isolates investigated**	**Origin**
*Flavobacterium branchiophilum*	1	(France)
*F. aquatile*	1	(France)
*F. aquidurense*	1	DSM18293
*F. columnare*	2	(France) (USA)
*F. frigidimaris*	1	(France)
*F. frixellicola*	1	(France)
*F. hercynium*	1	DSM18292
*F. hydatis*	1	DSM2063
*F. johnsoniae*	1	(France)
*F. limicola*	1	DSM15094
*F. pectinovorum*	1	DSM6368
*F. psychrolimnae*	1	(France)
*F. psychrophilum*	100	DSM3660 and isolates from BTF, BTL and RT
*F. succinicans*	1	DSM4002
*Flavobacterium* spp.	88	Water, tank swab and fish isolates from BTF and RT
*Chryseobacterium* spp.	17	Water and tank swabs
Other Aquatic Bacteria	11	Water, swab and fish isolates from BTF BTL and RT

*RT* rainbow trout, *BTF* brown trout fario; *BTL* brown trout lacustris.

### qPCR standards and spiked spleens

**Figure 1:**
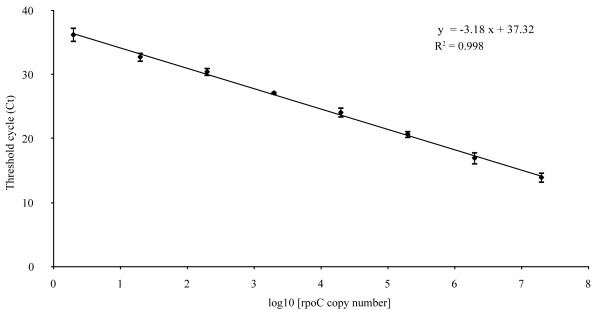
**Calibration of standards.** Each cycle threshold (Ct value) point corresponds to the mean of the 20 standards (each measured in triplicate) of samples. Regression coefficients for the 20 standards plotted: slope −3.18, intercept +37,32, R^2^: 0.998.

All qPCR standards and sample runs met the reliability criteria defined in the methods. We observed a good correlation between cycle threshold (Ct) values and quantifications of standards, with the slope of the linear regression curve over a 7-log range from 2 × 10^7^ to 2 × 10^0^*rpoC* gene copies being −3.18 (R^2^ = 0.998), indicating an efficiency of 106% (Figure 1). Purified, amplified fragment dilutions were therefore used for all successive quantifications as standards. The limit of detection (LOD) was 20 gene copies per reaction (LOD 100%). It was possible to amplify 2 *F. psychrophilum rpoC* gene copies per reaction in 90% of cases. This value is lower than the theoretical value reported by Bustin et al. [32], who concluded that the most sensitive LOD theoretically possible would be 3 copies per reaction, with a 95% chance of including at least 1 gene copy. The quantification limit (QL) was 10^3^ gene copies per reaction (QL 96%). This comparatively high value can be explained by losses during the DNA extraction procedure in samples with low bacteria concentrations.

**Figure 2:**
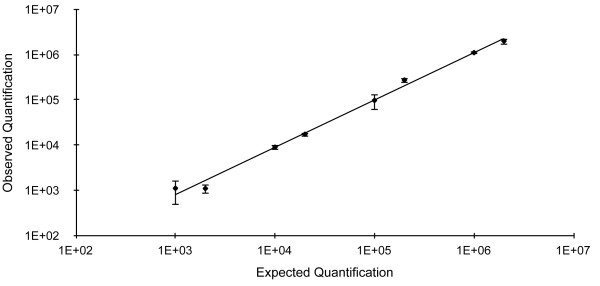
**Expected and observed *F. psychrophilum *cells.** Cell number detected in a mixture with *F. columnare* (10^7^, 10^4^, 10^3^ and 10^2^ cells per reaction) and *F. branchiophilum* (number of bacteria 10^6^, 10^4^, 10^3^ and 10^2^ cells per reaction). Slope: 1.0156, R^2^ = 0.9961.

qPCR showed a weak cross-reaction with the highest *F. branchiophilum* and *F. johnsoniae* pure DNA concentrations (respectively 10^6^ cells and 10^7^ cells per reaction, with a mean of 50 and 100 copies detected). This values, however, showed standard deviations >25% and were thus to be considered as negative according to the reliability check rules we adopted. To investigate cross-reaction with other DNA from fish pathogenic flavobacteria, qPCR was tested on mixtures of *F. psychrophilum* and *F. columnare or F. branchiophilum* DNA. Our qPCR showed a high specificity for *F. psychrophilum* and the agreement between observed and expected values of mixed samples was very good even at low copy numbers of the *F. psychrophilum rpoC* gene (Figure 2).

**Figure 3:**
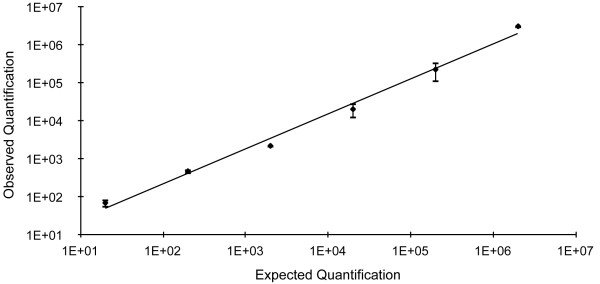
**Expected and observed *F. psychrophilum *cells in spiked spleens.** Concentrations of 5 *F. psychrophilum* isolates (from 2 × 10^1^ to 2 × 10^6^ cells per reaction), slope: 1.5678 and R^2^ = 0.9991.

*F. psychrophilum* could be reliably detected also in spiked spleens (linear results down to 20 cells per reaction, R^2^ = 0.9991). Quantification was reproducible without any observed interaction between spleen tissue DNA and the qPCR probe and primers (Figure 3).

### Detection and quantification of F. psychrophilum in environmental samples

No *F. psychrophilum* could be detected in any of the water samples by culture or FISH.

*F. psychrophilum*, however, could be discovered by qPCR in 7% of the inlet water samples and 53% of the tank water samples (LOD ≥ 20 copies, i.e. 66 *F. psychrophilum* cells/ml sampled) in a subset of 60 inlets and 60 water tanks samples from fish farms reporting at least one *F. psychrophilum* outbreak in 2009; a positive inlet was correlated with positive tank samples (n = 4) while no correspondence was observed in 29 farms, which had throughout positive tank water samples (min and max values: from 42 to 3,200 cells/ml) but negative inlet water. Values over the QL (3,300 *F. psychrophilum* cells/ml sampled) were observed only in 1 pair of inlet and tank water samples with values of 1.5 × 10^4^ ± 352 and 3.5 × 10^4^ ± 724 cells/ml (Table 2). Due to the comparatively high number of tank water samples testing positive for *F. psychrophilum* observed in the first subset of samples examined, we decided to screen all 2010 tank samples. Of the 85 tank water samples collected in 2010, however, only 8 (10%) were positive (range: 43 to 3,000 cells/ml) (Table 2).

**Table 2:** **Origin and percent of samples positive to *F. psychrophilum***

	**Origin**	**No. of samples**	**% Positive for *F. psychrophilum***	**% of samples quantified**	**Cells/ml**
Inlet and tank 2009					
Inlets	Ticino fish farms	60	7%	1.6%	73 to 1.5 × 10^4^
Tanks	Ticino fish farms	60	53%	1.6%	42 to 3.5 × 10^4^
2010					
Tanks	Swiss fish farms	85	10%	0%	43 to 3’000
Healthy carriers 2011, 2012	Swiss fish farms	43	80%	0%	0-400

In contrast to culture or FISH, *F. psychrophilum* was detected in healthy and quantified in infected fish by qPCR. *F. psychrophilum* densities in healthy individuals were well below the QL, in a range of 0 to 15,000 cells per spleen, whereas spleens from diseased fish contained bacterial densities over the QL, in a range of 7,000 to 7.7 × 10^8^ cells per spleen. Positive results by qPCR were reported for all spleens originating from the 4 outbreaks; FISH allowed detecting *F. psychrophilum* in all outbreaks while culture showed *F. psychrophilum* only in 3 outbreaks.

### Risk factors

**Figure 4:**
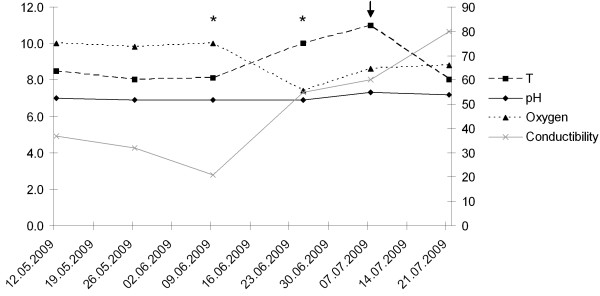
**Seasonal variation example.** Physicochemical parameters [primary y axis: temperature (T in °C), pH of water, oxygen concentration (mg/L); secondary y axis: conductibility (μ Siemens)] measured in a selected fish farm (Ticino, Switzerland) during 2009. Detection of the pathogen in the tank water samples started on 9 June 2009 (*), the arrows indicate a flavobacteriosis outbreak in brown trout fario.

We could not show any clear correlation between the presence of *F. psychrophilum* and the environmental parameters measured. We observed that the *F. psychrophilum* densities tended to increase and to cause outbreaks after changes in water parameters. For instance, a change in more than one ecological parameter tended to correlate with an outbreak or at least an increase of the number of *F. psychrophilum* in water (Figure 4). This observation, however, cannot be supported by any statistical analysis, because too few outbreaks could be analyzed during the study period.

## Discussion

This study shows that the qPCR assay developed is very sensitive and able to detect and quantify *F. psychrophilum* in water samples and fish spleens with no amplification of the other 130 non-target bacterial isolates.

In the water samples investigated, LOD was 20 *rpoC* gene copies per reaction and QL 10^3^ cells per reaction. The quantification limit was quite high: possibly random losses happened because of DNA uptake in columns during extraction of low cell concentrations. As DNA extraction from samples containing <1000 cells/μl was probably low, the quantification by qPCR was also not reliable. In a 16S rRNA gene *F. psychrophilum* qPCR recently described, quantification was based on the assumption that all isolates of *F. psychrophilum* have 6 repetitions of the 16S rRNA gene present in their genome [26]. This qPCR, however, needs to be adjusted for the number of 16S rRNA genes. It also showed to be less reliable by amplifying non-target DNA after ~30 cycles, while a qPCR based on the *rpoC* gene supplies direct quantification and is more reliable at low bacterial DNA concentrations. The *rpoC* gene is present in all *Flavobacterium* genomes so far investigated [30,33-36] and has already been used to identify clusters of species and species relatedness in taxonomy instead of 16 s rRNA [27,29]. While the 16S rRNA qPCR is doubtless more sensitive (down to 9 gene copies), we expect our qPCR to be more specific for *F. psychrophilum*. While we were developing and testing our qPCR, Marancik and Wiens [25] were developing a single copy gene PCR based on a sequence coding for a conserved *F. psychrophilum* protein with unknown function. They reported the limit of detection of their method to be 3.1 genome units per reaction, while for our qPCR it is approximately 20. On the other hand, their quantification limit in the spleen was approximately 500 bacteria in 1.5 μl of a 200 μl DNA elution, while our limit was 20 bacteria in 2 μl of reaction mixture. In addition, while Marancik and Wiens [35] tested their qPCR only against a limited number of non-target organisms and only under laboratory conditions, we challenged our qPCR against strains of different fish pathogens and of bacterial genera normally present in water. In addition, we tried to carry out our testing under conditions reflecting a real-life situation where bacterial species (including other fish pathogens) and substances (antibiotics, minerals, humic acids) are normally present and can interfere with the target organism detection and quantification. Overall, however, we would expect Marancik & Wiens’ and our methods to be roughly comparable, although our quantification limits in the spleen is better and we were able to demonstrate the applicability of our technique also on water samples from fish farms.

Cross-reactions with other species belonging to the same genus were not observed in *in silico* testing of primers against the entire genome of *F. branchiophilum*, *F. columnare*, *F. indicum* and *F. johnsoniae*. When the qPCR was used on mixed samples of *F. psychrophilum* with *F. columnare* and *F. branchiophilum* no cross-reaction was observed. In addition, quantification in spiked spleens gave linear results down to a concentration of 20 bacteria per reaction. In our study we used rather low concentrations of bacteria to spike spleen tissues (10^2^ cells/mg), as opposed to other studies in which higher bacterial loads were used. We thus conclude that the qPCR presented here is highly specific for the target organism.

*F. psychrophilum* seem to be present only in few samples at detectable values, tanks being more often colonized than inlet waters. 53% and 10% of tank water samples collected in the fish farms respectively during the years 2009 and 2010 were positive for *F. psychrophilum* by qPCR. Data seem thus to suggest a high prevalence of the pathogen in 2009, with a regression in 2010, but this is most likely a consequence of the different sampling strategies adopted in the two seasons. In 2009, in fact, we screened only fish farms in Ticino where outbreaks of *F. psychrophilum* occurred, whereas in 2010 all Swiss fish farms under investigation were screened independently of any outbreaks diagnosis. We also used only 15 ml water samples, whereas increasing the sample volume may also increase the probability to detect *F. psychrophilum* in environmental water samples. In addition, this was only a preliminary study to test the technique and its limits in natural field conditions: the study was neither planned nor powered to allow drawing any conclusions or making any interpretations about the disease distribution.

Unfortunately little is known about the pathogen in its environment and about its mode of transmission. We suggest that *F. psychrophilum* could be present and replicate in the tank (in both, fish and organic layer) and diffuse in the water [37], where favourable ecological conditions would allow colonization/infection of other fishes.

*F. psychrophilum* detection by qPCR in the spleen of diseased and symptomless fishes suggests that the pathogen may have already been present in the spleen of symptomless fish at densities below QL but above LOD. Marancik and Wiens [25] report similar results using their qPCR, which detected the presence of *F. psychrophilum* in few symptomless carriers that had been infected with the pathogen. In contrast, no infection was recorded prior to sampling of healthy-looking fishes in our study. Thus, *F. psychrophilum* is apparently able to colonize and live asymptomatically in the spleen, where it is inactive until favorable environment conditions and a weakening of the fish immune system allow this opportunistic pathogen to multiply, spread in the fish and eventually in the whole fish population. During outbreaks, fish spleen harbored higher amounts of the pathogen, at concentrations markedly higher than the QL. Healthy, colonized fish may thus act as reservoirs for infection: in our opinion, this is a valid assumption, because another study has demonstrated the presence of this pathogen in eggs and ovarian fluids [38]. Further investigations, however, are needed to assess the mode of transmission and ecology of this species.

qPCR detected and quantified *F. psychrophilum* in all 4 *F. psychrophilum* outbreaks investigated in this study; 13 of 15 qPCR values were higher than LOD, and in 8 cases higher than the QL. FISH could also detect all outbreaks, while culture methods could detect only 3 outbreaks and one was incorrectly recorded as negative.

Changes in water temperature (e.g. a temperature variation of 4°C), oxygen availability in water, pH and conductibility could lead to a disease outbreak. In our study, changes in two or more parameters seemed to correlate with the detection of *F. psychrophilum* in the water. Factors that decrease host immune response are often crucial for the establishment of an infection by opportunistic pathogens [39,40]. Seasonality, for instance, was found to impact the Rainbow trout immune system due to pathogen density being lower in winter than in summer. Moreover, differences between winter and summer water temperatures may significantly change red blood cells counts in fish [41]. Different studies suggest also population densities in tanks as a potential risk factor [42-45]. Karvoven et al. [43] reported a positive correlation between temperature and onset of *F. columnare* infections, while a negative correlation was found between the presence of the flagellate *Ichthyobodo necator*, the causal agent of costiasis, and temperature. *I. necator* was also isolated from fish infected by *F. psychrophilum*[46]. Unfortunately, our observations on potential risk factors are restricted to four documented outbreaks only. It is therefore not possible to carry out any statistical analysis to describe potential interactions between factors and to quantify the importance of each factor for the establishment of the infection.

## Conclusions

This study has shown that qPCR using the *rpoC* gene could be used as a reliable, specific diagnostic tool to detect and quantify *F. psychrophilum* colonisations and infections. This technique could be used to screen for the presence of the pathogen in fish farms in order to prevent devastating outbreaks. qPCR could also be applied in investigations of vertical pathogen transmission [15,38], to perform studies of risk factors including different stress conditions, and to check for outbreaks due to network structures among fish farms [47]. The symptomless presence of *F. psychrophilum* we have observed in some fish samples indicates that the survival of the pathogen may contribute to a significant risk for outbreaks caused by fish trade, with healthy carriers coming into contact with other individuals from different origins.

## Methods

### Sampling strategy

Water samples were collected in 2009 and in 2010 from the inlets and fish tanks of 22 independent Swiss fish farms. Inlet water flew directly from the river into separate tanks; the water volume ranged from 2 to 105 m^3^. The water flow was continuous. The detailed sampling structure is described in Table 2.

During 2009, water and different fish species were sampled every second week in 4 fish farms located in the Ticino Canton (Switzerland) (60 sampling actions).

In 2010, sampling was carried out in 22 fish farms all over Switzerland at 3 different periods (85 sampling actions). The first was in winter shortly before fishes started hatching (only water), the second was carried out 6 and the third 12 weeks after hatching and when fishes started feeding. At each sampling date six fishes [Rainbow trout (*Oncorhinchus mykiss*) and brown trout (S*almo trutta fario* and *Salmo trutta lacustris*)] were collected concomitantly with two water samples, one from the inlet and one from the tank. 50 ml of water were collected in 50 ml Falcon tubes (Becton Dickinson BD, Switzerland), while fishes were collected in a container with water and brought back to the laboratory within 24 h after collection in refrigerating bags. Plating and fixation of water samples were carried out immediately upon arrival in the laboratory.

Population density of fishes in the tanks, physical (temperature, water conductibility, oxygen saturation, water volume) and chemical (disinfectant and antibiotic use) water parameters were recorded directly at the fish farm.

In the laboratory, 100 μl of water collected were plated on Cytophaga enriched Agar Medium (CAM, medium 1133 DSMZ: 0.2% tryptone, 0.05% beef extract, 0.05% yeast extract, 0.02% sodium acetate, 1.5% agar). All plates were incubated at 15°C during 5 to 10 days. Yellow colonies (i.e. putative flavobacteria) were transferred onto fresh plates and screened with a *Flavobacterium* spp. and *F. psychrophilum* specific FISH [16]. Pure cultures of *Flavobacterium* spp. and *F. psychrophilum* were conserved at −80°C in 1 ml skimmed milk (Becton Dickinson, Switzerland) supplemented with 10% bovine serum and 20% glycerol.

Fixation of water samples was carried out according to Tonolla et al. [48] with the following modifications: 15 ml of each water sample were filtered with a Millipore filtration system (Merck Millipore) with 3.0 μm mesh size filters overlaid with 0.2 μm mesh size filters. Each sample was covered with 4% Paraformaldehyde Fixation Buffer (PBS: 0.13 M NaCl, 7 mM Na_2_-HPO_4_, 3 mM NaH_2_PO_4_, pH 7.2) for 30 min and then washed twice with 1× Phosphate Buffered Saline (PBS). The overlay filters were transferred into plastic bags; 600 μl of a 50% PBS-ethanol solution were added, the bags sealed and bacteria re-suspended by slightly rubbing the filter between thumb and forefinger. The suspension was then transferred into a 1.5 ml Eppendorf tube and stored at −20°C until DNA extraction. The DNeasy Blood & Tissue Kit (QIAGEN - Switzerland) was used for DNA extraction of all fixed water samples.

For pathogen detection in animals, fish collected were killed by immersion in 0.01% benzocaine followed by section of the vertebral column. Spleen of rainbow trout, brown trout fario and brown trout lacustris were homogenized separately in 200 μl of sterile water. 190 μl of the homogenates were plated on CAM medium and incubated at 15°C for 5 to 10 days while the remaining 10 μl were used for FISH [16].

Approval for animal experiments and water collection was obtained from the Federal Veterinary Office (FVO, Switzerland) and the Ticino Cantonal Veterinary Office (Authorization 03/2010 and 04/2010).

### Identification of colonies and diagnosis of outbreaks by FISH

Identification of flavobacteria in general and *F. psychrophilum* in particular was carried out using a published FlSH protocol [16]. *F. psychrophilum* (DSM 3660), environmental *Flavobacterium* spp. and *Chryseobacterium* spp. isolates were used as positive and negative controls.

### rpoC qPCR design and test of primers

DNA was extracted using InstaGene kit [Bio-Rad, Hercules (CA), USA]. Partial DNA dependent β’ subunit RNA polymerase (*rpoC*) gene sequences were amplified based on the RNA polymerase β’ subunit primers sequences described by Griffiths et al. [49] with the addition of sequence tags UP1s and UP2sr (rpoC_F 5’- GAAGTCATCATGACCGTTCTGCAATHGGNGARCCNGGNACNCA-3’ and rpoC_R 5’- AGCAGGGTACGGATGTGCGAGCCGGNARNCCNCCNGTDATRTC-3’; synthesized by Microsynth, Switzerland) to increase sequencing performance [50]. The PCR reaction was carried out in a total volume of 50 μl using 2.5 U HotStarTaq DNA Polymerase (QIAGEN-Switzerland), 7 mM MgCl_2_, PCR Buffer 1X (QIAGEN-Switzerland), 0.2 mM dNTP (Roche, Switzerland), 0.2 μM of each forward and reverse primer, and 5 μl of InstaGene DNA extract. The thermal cycle started with 15 min HotStarTaq activation at 95°C followed by 36 cycles of 1 min at 94°C, 90 s at 55°C, 1 min at 72°C and eventually an elongation cycle of 7 min at 72°C.

Sequences (GenBank access numbers JX657163- JX657284) obtained from the rpoC gene general PCR were aligned using MEGA4 [51] and screened for a conserved species-specific fragment that would be used to design a set of primers and a TaqMan probe targeting specifically *F. psychrophilum*. Primers F.psychro_P1F 5’-GAAGATGGAGAAGGTAATTTAGTTGATATT-3’, F. psychro_P1R 5’- CAAATAACATCTCCTTTTTCTACAACTTGA-3’ and a minor groove binder (MGB), and probe F. psychrophilum_probe 5’- AAACGGGTATTC TTCTTGCTACA -3’ (Applied Biosystems) labeled with FAM were tested *in silico*[52] and with BLAST (Basic local alignment search tool [53]). The primers amplified a fragment of 164 bp. PCR was carried out in a final volume of 25 μl containing 1X Taq PCR Master Mix Kit (QIAGEN, Switzerland), 0.3 μM primers F. psychro_P1F and F. psychro_P1R, and 2.5 μl of genomic DNA. Conditions for amplification were 94°C for 1 min followed by 35 cycles of 94°C for 30 s, 56°C for 35 s and 72°C for 30 s, with a final elongation cycle of 7 min at 72°C.

DNA of *F. psychrophilum*, *Flavobacterium* spp. and other bacterial species isolated from soil, water and fish were used to test sensitivity and specificity of the primers. All tested bacteria and their origin are listed in Table 1.

### qPCR cycling parameters

The qPCR was carried out in a final volume of 20 μl containing 1× TaqMan Environmental Master Mix v.2.0 (Applied Biosystems), 0.9 μM of each primer, 0.2 μM of *F. psychrophilum* probe, 1X of internal control Exo IPC Mix, 1× of IC DNA (TaqMan Univ. MMix w Exog IntPostC, Applied Biosystems), and 2 μl of template DNA. An internal control was added to each reaction to check for PCR inhibitors. The run consisted of two cycles at 50°C for 2 min and 95°C for 10 min, followed by 40 cycles at 95°C for 15 s and 60°C for 1 min. All assays were carried out in triplicates. Water was used as negative control and series of quantified DNA dilutions as standards.

### Preparation of standards

*F. psychrophilum* DNA was amplified by PCR with primers F. psychroP1F and F.psychroP1R. The products were purified with PCR clean-up NucleoSpin® ExtractII (Macherey-Nagel, Germany) and quantified with a Nanodrop spectrophotometer (ND1000, Witek, Switzerland). The total amount of DNA measured was divided by 1.797 × 10^−7^ pg [the weight of one *rpoC* fragment (164 bp) [54-56]]. The result was an estimate of the number of gene copies in 1 μl of purified product. Serial dilutions from 1 × 10^7^ to 1 × 10^0^ copies/μl of amplified DNA were used to calculate the Limit of Detection (LOD) of the qPCR and as quantitative standards for further analyses.

Serial 10-fold dilutions were made starting from *F. psychrophilum* suspensions [Optical Density (OD_595_) 0.3 ± 0.02] corresponding to (3 × 10^9^) ± (7 × 10^8^) cells/ml [16]. Each suspension was extracted with DNeasy Blood & Tissue Kit (QIAGEN - Switzerland) and used to determine the quantification limit (QL).

### Limit of detection and quantification limit

Calibration curves were obtained by plotting cycle time (Ct) values against log_10_ (gene copies number). The coefficients of regressions as well as the R^2^ values were calculated. The LOD was calculated using a serial dilution from 2 × 10^7^ to 2 × 10^0^ amplified fragments per reaction of 20 *F. psychrophilum* amplified DNA standards.

Suspensions of 24 *F. psychrophilum* isolates (serial dilutions from 2 × 10^4^ to 2 × 10^−1^ cells per reaction) were analyzed to determine the QL. Genomic DNA standards from bacteria suspensions were used to check the reliability of the quantification.

qPCR specificity and potential cross-amplifications with other *Flavobacterium* spp. were checked using dilutions of DNA extracted from *F. branchiophilum* (concentrations: 10^6^ and 10^5^ cells per reaction), *F. columnare*, *F. johnsoniae*, *F. psychrolimnae*, *F. fryxellicola*, *Flavobacterium* sp. and *Chryseobacterium* sp. (concentrations: 10^7^ and 10^6^ cells per reaction). In addition, diluted DNA samples of *F. columnare* (10^7^, 10^4^, 10^3^, 10^2^ cells per reaction) and *F. branchiophilum* (10^6^, 10^4^, 10^3^, 10^2^ cells per reaction) were mixed with decreasing concentrations of *F. psychrophilum* DNA (from 10^6^ to 10^3^ cells per reaction).

### Reliability check

For the results of the qPCR to be reliable, the coefficient of the standards regression had to be in the range −3.6 – -3.0 (Applied Biosystems, manufacturer’s instructions for qPCR), the coefficient of variation of quantification within each standard and sample in triplicates <25% and the non target control (water) had to show no amplification within the run [54,57].

### qPCR of spleen samples

Spleens of diseased and symptomless rainbow trout and brown trout were gathered during 2011 and 2012 in the Ticino fish farms and treated as described before. Fish were considered healthy when they showed no disease symptoms and, additionally, no signs of infection or extraordinary mortality were reported in the fish farm.

In total 15 rainbow and brown trout spleens were collected and analyzed during 4 outbreaks while 43 spleens from symptomless fish (rainbow and brown trout) were collected in 2 different fish farms showing no sign of infection.

Spleens from symptomless fish were removed, weight calibrates and stored at −20°C until further processing. Mean spleen weight was 0.013 ± 0.007 g for rainbow trout and 0.007 ± 0.002 g for brown trout.

At the time of the experiments, spleens from healthy fishes were thawed and homogenized in 200 μl of sterile water. 100 μl of the suspension were spiked with known amounts of *F. psychrophilum* (10^6^ to 10^1^ cells per reaction) to a final volume of 100 μl and extracted using DNeasy Blood & Tissue Kit (QIAGEN). The remaining 100 μl were used as controls in FISH and DNA extraction for *F. psychrophilum* qPCR screening and quantification purpose.

Spleens from diseased fish were used to quantify levels of infection under real-life conditions. They were removed and homogenized in 200 μl of sterile water. It was, however, not possible to weight them. 90 μl of the spleen homogenates were plated on CAM and incubated at 15°C for 5 to 10 days while 10 μl were analysed using FISH with the PanFlavo and *F. psychrophilum* probes [16]. DNA was extracted from the remaining 100 μl.

### Statistical analysis

Primer specificity (SP) and sensitivity (SE) as well as positive and negative predicted values were assessed by standard PCR. The efficiency of qPCR was calculated as E = 10^-1/slope^-1. A linear regression was used to calculate the LOD and the QL at the fifth percentile of all analyzed samples correctly detected (LOD) or quantified (QL) by the technique using SPSS Statistics for Windows, Version 20.0 (IBM Corp., Armonk, NY).

## Competing interests

The authors declare that they have no competing interests.

## Authors’ contributions

NS conceived the study, carried out the Taqman quantitative PCR, analyzed the results and drafted the manuscript. OP participated in the design of the study, analyzed the results and helped in writing the manuscript. HS and TW helped to design the study and to draft the manuscript. All authors read and approved the final version.
